# Effects of Growth Stage on the Characterization of Enterotoxin A-Producing *Staphylococcus aureus*-Derived Membrane Vesicles

**DOI:** 10.3390/microorganisms10030574

**Published:** 2022-03-06

**Authors:** Yuka Yamanashi, Yuko Shimamura, Haruka Sasahara, Misaki Komuro, Kuniaki Sasaki, Yasujiro Morimitsu, Shuichi Masuda

**Affiliations:** 1 School of Food and Nutritional Sciences, University of Shizuoka, 52-1 Yada, Suruga-ku, Shizuoka 422-8526, Japan; s20205@u-shizuoka-ken.ac.jp (Y.Y.); shimamura@u-shizuoka-ken.ac.jp (Y.S.); s17210@u-shizuoka-ken.ac.jp (H.S.); 2 Faculty of Science and Engineering, Iwate University, Ueda 3-18-8, Morioka, Iwate 020-8550, Japan; komuro@iwate-u.ac.jp (M.K.); kuniaki@iwate-u.ac.jp (K.S.); 3 Department of Nutrition and Food Science, Ochanomizu University, 2-1-1 Otsuka, Bunkyo-ku, Tokyo 112-8610, Japan; morimitsu.yasujiro@ocha.ac.jp

**Keywords:** *Staphylococcus aureus*, membrane vesicles, staphylococcal enterotoxin A, growth stages, inflammation

## Abstract

Virulence factors, such as staphylococcal enterotoxin A (SEA), are contained within membrane vesicles (MVs) in the cell membrane of *Staphylococcus aureus*. In this study, the effects of the growth stage on quantitative and qualitative changes in the components contained in the MVs of *S. aureus* SEA-producing strains were examined. Changes in the expression levels of *S. aureus* genes were examined at each growth stage; phenol-soluble modulin (PSM) gene reached a maximum after 8 h, and the expression of cell membrane-related genes was decreased after 6 h. Based on these gene expression patterns, MVs were prepared at 6, 17, and 24 h. The particle size of MVs did not change depending on the growth stage. MVs prepared after culture for 17 h maintained their particle size when stored at 23 °C. The amount of SEA in the culture supernatant and MVs were not correlated. Bifunctional autolysin, a protein involved in cell wall biosynthesis/degradation, was increased in MVs at 17 h. The expression pattern of inflammation-related genes in human adult low calcium high temperature (HaCaT) cells induced by MVs was different for each growth stage. The inclusion components of *S. aureus*-derived MVs are selective, depend on the stage of growth, and may play an important role in toxicity.

## 1. Introduction

*Staphylococcus aureus* produces a variety of toxins and extracellular proteins, including staphylococcal enterotoxins (SEs), and causes various diseases, such as sepsis [1], pneumonia, and skin infections [2]. Staphylococcal food poisoning, which is a typical disease, is caused by the ingestion of SEs released from *S. aureus* growing in food [3]. Although more than 20 types of SE have been reported, there are many cases of food poisoning due to enterotoxin A (SEA) worldwide [4]. Most SE production is regulated by the accessory gene regulator (*agr*), but the regulatory mechanism of SEA is at least partially associated with the lysogenic phage life cycle [5,6,7]. Therefore, SEA production is not expected to correlate with culture time or the bacterial count. The genes encoding SEA are on the bacteriophage genome and their life cycle is characterized by two stages: lysogenic and lytic [8].

In addition, the virulence factors of *S. aureus* are also contained in membrane vesicles (MVs) from about 20 to 500 nm in size; these are composed of the cell membrane of the bacterium itself [9]. MVs function as transporters of various substances, including virulence factors, adhesion factors, DNA, RNA, intercellular communication signaling substances, and immunomodulators [10]. The proteomic analysis of MVs in *S. aureus* revealed that MVs contain extracellular and cytoplasmic proteins, as well as multiple virulence factors [11]. In Gram-positive bacteria, MVs formation can be caused by phage-derived endolysin breaking down the cell wall, and the cell membrane protrudes from the hole resulting from peptidoglycan. Such MVs formation often causes cell death and is called bubbling cell death [12]. Phenol-soluble modulin (PSM) peptides, which are a family of cytolytic peptide toxins, promote the release of membrane vesicles from the cell membrane of *S. aureus* [13]. The PSM family contains short amphipathic α-helix peptides. The PSM family has been identified to have seven complexes containing PSMα1–α4, PSMβ1–β2, and δ toxins, and has multiple roles in *S. aureus* infection [14,15]. It was reported that significant amounts of lipoproteins were released into the culture supernatant only when PSM was strongly expressed [16]. Lipoprotein release is regulated by the *agr* quorum sensing system, which regulates the expression of PSM peptides, which release lipoproteins [16]. As MVs have a different protein composition than bacterial cells [13], it is considered that there is a selective transport mechanism for inclusion components of MVs. The presence of virulence factors, such as SEA, in MVs, results in high local concentrations in foods and host cells, which may contribute to the more efficient development of food poisoning and diseases. There has been no study of the relationship between the production level of SEA and the amount of SEA included in MVs.

Although the composition and release mode of MVs have not been clarified, the introduction of curvature into the outer membrane due to repulsion between anionic charges on the cell membrane surface is thought to be one of the factors causing MVs formation [17]. However, the relationship between the membrane stability of MVs and the changes in cell membrane components has not been clarified. This study focused on the changes in the expression levels of the following four cell membrane-related genes: poly(glycerophosphate chain) d-alanine transfer protein (Dlt), multiple peptide resistance factor (MprF), phosphatidylglycerol synthase (PgsA), and cardiolipin synthases (Cls2). The Dlt modifies both lipoteichoic acid and mural teichoic acid with d-alanine, reducing the density of negative charges on the cell wall. It was suggested that Gram-positive cells can regulate the quorum sensing system under stress by regulating the density of the negative charges on the cell wall [18]. MrpF is a membrane protein that catalyzes the modification of negatively charged phosphatidylglycerol with L-lysine and affects the susceptibility of *S. aureus* to cationic antibiotics [19]. The main components of bacterial membranes contain various types of phospholipids, such as phosphatidylethanolamine, phosphatidylglycerol (PG), and cardiolipin (CL) [20,21]. The enzymes PgsA and Cls2 are involved in PG synthesis and CL synthesis, respectively [22,23,24].

It has been suggested that MVs play a role in the delivery of *S. aureus*-derived effector molecules to host cells and that molecules packaged in MVs contribute to the keratinocyte pro-inflammatory response in vitro [25]. It was reported that *S. aureus*-derived MVs stimulated the expression of pro-inflammatory cytokines (interleukin (IL)-1β, IL-6, and tumor necrosis factor (TNF)-α) and chemokine (IL-8 and monocyte chemoattractant protein-1 (MCP-1)) genes in a spontaneously immortalized human epidermal keratinocyte strain, human adult low calcium high temperature (HaCaT) cells [26]. The phosphatase and tensin homolog deleted on chromosome 10 (PTEN) is a dual phosphatase that dephosphorylates lipids and proteins [27]. It has been reported that the suppression of PTEN phosphatase activity leads to enhanced wound repair [28]. In vitro studies have shown that the inhibition of miR-155 reduces keratinocyte proliferation and increases PTEN gene expression [29]. However, the effect of the growth stages of *S. aureus* on the components included in MVs and the pro-inflammatory response and proliferation of HaCaT cells has not been clarified.

The purpose of this study was to clarify the effect of growth stages on the quantitative and qualitative changes in the components included in the MVs of SEA-producing strains. Virulence factors in the culture supernatant are considered to increase with the growth stage. On the other hand, in MVs, considering that release and destruction occur at the same time and that there is an inclusion mechanism of selective virulence factors, it is possible that the properties of MVs may differ at each growth stage. To evaluate the gene expression pattern of SEA-producing strains from different growth stages, the changes over time in the expression levels of the SEA gene (*sea*), *agr* quorum sensing control gene (RNAIII), PSM gene (*psm*), and cell membrane-related genes (*dlt*D, *mpr*F, *pgs*A, and *cls*2) of the SEA-producing strain were examined. Based on these gene expression patterns, MVs were prepared from the culture supernatant of a SEA-producing strain at 37 °C for 6 (log phase), 17 (stationary phase), and 24 (phase of decline) h. The difference in the particle size of MVs and the proteins contained in MVs, such as SEA, at each incubation time obtained, were examined. In addition, MVs at each growth stage were added to HaCaT cells, and the pro-inflammatory response and proliferation were analyzed.

## 2. Materials and Methods

### 2.1. Bacterial Strain and Culture Conditions

*S. aureus* C-29 (SEA-producing strain) was isolated from human hands [30]. *S. aureus* C-29 was inoculated into brain heart infusion (BHI) broth and incubated at 37 °C for 24 h with shaking (190 rpm). After 24 h, the culture solution (30 µL) was inoculated into 3 mL of BHI broth and incubated at 37 °C for 18 h with shaking. The obtained culture solution was centrifuged (11,600× *g*, 3 min) and the supernatant was removed. The bacteria were washed twice with phosphate-buffered saline (PBS) and resuspended in PBS. This bacterial suspension was used as the bacterial inoculum.

### 2.2. Measurement of Gene Expression in Enterotoxin A-Producing Staphylococcus aureus

The bacterial inoculum (70 µL) of *S. aureus* C-29 was inoculated into 7 mL of BHI broth. This suspension (200 µL) was added to 96-well plates and incubated at 37 °C for 2, 4, 6, 8, 10, 15, and 17 h. The culture solution obtained at each time was centrifuged (11,600× *g*, 3 min), the supernatant was removed, and the pellet was recovered. RNA was extracted from the pellet using RiboPure^TM^-Bacteria (Invitrogen, Carlsbad, CA, USA) in accordance with the manufacturer’s instructions. The total RNA purity and concentration was measured with a K2800 nucleic acid analyzer (Beijing Kaiao Technology Development Co., Ltd., Beijing, China). The cDNA was synthesized using the PrimeScript RT reagent kit (TaKaRa Bio Inc., Shiga, Japan) and stored at −20 °C until use. Real-time reverse transcription (RT)-PCR was performed using the Thermal Cycler Dice^®^ Real-Time System II (TaKaRa Bio Inc., Shiga, Japan). The 16S rRNA gene was used as an internal standard to correct the amount of mRNA between samples. The fold change value with a logarithm transformation [log 2 (2^–ΔΔCt^)] was calculated. The sequences of the primers used are shown in Table 1.

### 2.3. Isolation of Membrane Vesicles (MVs)

The bacterial inoculum (500 µL) of *S. aureus* C-29 was inoculated into 250 mL of BHI broth and incubated at 37 °C for 6, 17, and 24 h with shaking (110 rpm). The culture solution obtained at each time was transferred to 50 mL polypropylene sterile disposable centrifuge tubes and centrifuged (3000× *g*, 15 min). After the bacterial cell pellet was removed, the supernatant was filtered through a 0.2 µm filter (Advantec Toyo Co., Ltd., Tokyo, Japan). The filtrate was transferred to Amicon Ultra centrifugal filter devices 100K (Merck, Darmstadt, Germany) and centrifuged (4000× *g*, 30 min). A 100 kDa cutoff (>100 kDa) concentrate was added to the ultracentrifugation tubes (11PA thick-walled tubes, Hitachi Koki Co., Ltd., Tokyo, Japan) and ultracentrifuged (150,000× *g*, 4 °C, 3 h) using the Himac CP-WX Series separation ultracentrifuge (Hitachi Koki Co., Ltd., Tokyo, Japan). The pellet was resuspended in 100 µL of PBS, and the amount of protein was measured using a micro-spectrophotometer (Nikko-Hansen Co., Ltd., Osaka, Japan). The isolated MVs were stored at −20 °C until use. Unless otherwise stated, the MVs were used as undiluted.

### 2.4. Measurement of Particle Size Distribution

Each isolated MV (30 µL) was diluted with 970 µL of PBS. The particle size distribution of MVs from *S. aureus* C-29 was analyzed by dynamic light scattering (DSL; Zetasizer Ultra ZS Particle Analyzer, Malvern, UK). Although scattering intensity, number, and volume standards can be used to evaluate particle size, number standards often used for comparison with electron microscope images were used.

### 2.5. Transmission Electron Microscopy (TEM) Analysis

Each isolated MV was diluted 5–20 times with PBS. The dilute MV solution was stained negative with 1% aqueous uranyl acetate and added dropwise to a microgrid. After air-drying, the MVs were observed with a JEM-2100 microscope (JEOL, Tokyo, Japan) at an acceleration voltage of 80 kV.

### 2.6. Evaluation of the Storage Stability of MVs

Considering the presence of *S. aureus*-derived MVs in the natural environment, a long-term incubation test at 23 °C (assuming room temperature) was conducted to investigate how long the MVs could retain their shape. Each isolated MV (30 µL) was diluted with 970 µL of PBS and stored in the dark at 23 °C for 0, 2, or 5 days, or 1 week. After each storage period, the diluted MV solution was filtered through a 0.2 µm filter (Advantec Toyo Co., Ltd., Tokyo, Japan). The particle size distribution of MVs was analyzed by DSL (Zetasizer Ultra ZS Particle Analyzer, Malvern, UK) using the method described in Section 2.4.

### 2.7. Detection of SEA Using Western Blot Analysis

Each isolated MV was boiled for 5 min in sample buffer (1 M Tris–HCl (pH 6.8), 10% SDS, 0.5 mg/mL of bromophenol blue, 25% 2-mercaptoethanol, and 20% glycerol). SEA (>95% purity; Toxin Technology, Sarasota, FL, USA) was used for the loading control (final concentration was 100 ng/mL). The proteins were separated by SDS-PAGE using a 15% polyacrylamide gel (20 mA for 90 min). All Blue Regular Range Protein Marker PM1500 (Cosmo Bio, Tokyo, Japan) was used as the molecular weight marker. The separated proteins were transferred from the gel onto a PVDF membrane using a semi-dry transfer unit (400 mA for 90 min; Oriental Instruments, Co., Ltd., Tokyo, Japan). After transfer, the PVDF membrane was immersed into the blocking solution (5% skim milk powder for blotting; FUJIFILM Wako Pure Chemical Corporation, Osaka, Japan) for 90 min at room temperature. After blocking, the PVDF membrane was immersed in a primary antibody solution of anti-rabbit SEA (Sigma-Aldrich, Saint Louis, MO, USA) diluted 1:5000 (*v*/*v*) with PBS and stored overnight at 4 °C. Then, the PVDF membrane was washed three times for 5 min in wash solution (20× Wash Solution Concentrate; KPL, Gaithersburg, MD, USA). The washed PVDF membrane was immersed in a secondary antibody dilution of anti-rabbit peroxidase conjugate (KPL) diluted 1:1000 (*v/v*) with PBS for 90 min at room temperature. The PVDF membrane was washed for 5 min in wash solution and twice in PBS for 5 min. The PVDF membrane was immersed in a substrate solution (BCIP/NBT, KPL, Gaithersburg, MD, USA) and incubated at room temperature for 30 min for the color to develop. After obtaining a suitably stained image, the membrane was washed with water, air-dried, and the band on the membrane was quantified using image analysis software (ImageJ; National Institutes of Health, Bethesda, MA, USA).

### 2.8. SDS-PAGE and Liquid Chromatography-Tandem Mass Spectrometry (LC–MS/MS)

The patterns of the protein bands of MVs were examined by SDS-PAGE, as described above. The electrophoresed 15% polyacrylamide gel was stained in 0.1% Coomassie brilliant blue in 50% methanol and 7% acetic acid for 30 min and then destained in 7% acetic acid and 10% methanol. Nano-LC–MS/MS analyses of samples subjected to in-gel digestion with trypsin were performed by Japan Proteomics Co., Ltd. (Sendai, Japan).

### 2.9. MV-Induced Inflammation-Related Mediator Release in Rat Basophilic Leukemia (RBL-2H3) Cells

RBL-2H3 cells were cultured in Dulbecco’s modified Eagle’s medium (DMEM; Thermo Fisher Scientific, Waltham, MA, USA) supplemented with 20% fetal bovine serum (Thermo Fisher Scientific, Waltham, MA, USA), ×1 Glutamax (×100, Thermo Fisher Scientific, Waltham, MA, USA), 100 units/mL of penicillin, and 100 μg/mL of streptomycin (Thermo Fisher Scientific, Waltham, MA, USA) at 37 °C under 5% CO_2_. RBL-2H3 cells were seeded in a 24-well plate at 8.0 × 10^4^ cells/well and sensitized with 0.05 µg/mL of anti-DNP IgE (Sigma-Aldrich, Saint Louis, MO, USA) at 37 °C in an atmosphere of 5% CO_2_ for 24 h. The cells were washed twice with PBS (Thermo Fisher Scientific, Waltham, MA, USA). After washing, 180 µL of release mixture (116.9 mM NaCl, 5.4 mM KCl, 0.8 mM Mg_2_SO_4_⋅7H_2_O, 5.6 mM glucose, 25 mM HEPES, 2.0 mM CaCl_2_, and 1.0 mg/mL BSA, pH 7.7) was added to all wells, other than the well containing the positive control, and incubated at 37 °C under 5% CO_2_ for 10 min. Isolated MVs (20 µL) were added to all wells other than the positive control and incubated at 37 °C in 5% CO_2_ for 1 h. BHI broth (20 µL) was used as the negative control. A solution of 0.4% digitonin (FUJIFILM Wako Pure Chemical Corporation, Osaka, Japan) (200 µL) was added to the positive control well and incubated in the same manner. After the incubation, 20 μL of DNP BSA (15.2 ng/mL; Cosmo Bio, Tokyo, Japan) was added to the well and incubated at 37 °C in 5% CO_2_ for 30 min to stimulate degranulation of the cells. The reaction was stopped by immersion in an ice bath for 10 min. The supernatant (50 µL) was transferred to a 96-well plate and 50 µL of 5 mM *p*-nitrophenyl-*N*-acetyl-β-d-glucosaminide in 0.05 M citrate buffer (pH 4.5) was added and incubated at 37 °C for 30 min. The reaction was stopped by the addition of 200 μL of stop buffer (0.1 M Na_2_CO_3_ and 0.1 M NaHCO_3_, pH 10.0). The absorbance at 405 nm was measured with a microplate reader. The β-hexosaminidase release rate (%) from each MVs was calculated from the following equation:

(1) $$\beta \text{-}\mathrm{Hexosaminidase}\;\mathrm{release}\;\mathrm{rate}\;\left(\%\right)=\frac{A\;\left[\mathrm{sample}\right]-A\;\left[\mathrm{control}\;\left({\mathrm{DNPBSA}}^{-}\right)\right]}{A\;\left[\mathrm{digitonin}\right]-A\;\left[\mathrm{control}\;\left({\mathrm{DNPBSA}}^{-}\right)\right]}$$

### 2.10. Expression Level of MV-Induced Inflammation-Related Genes in HaCaT Cells

HaCaT cells were cultured in MG-30 (DMEM High Glucose ready-to-use, with serum, CLS Cell Lines Service, Eppelheim, Germany) supplemented with 50 units/mL of penicillin and 50 μg/mL of streptomycin (Thermo Fisher Scientific, Waltham, MA, USA) at 37 °C under 5% CO_2_. HaCaT cells were seeded at 4.0 × 10^4^ cells/well in a 96-well plate and cultured at 37 °C in 5% CO_2_ for 24 h. Isolated MVs (10 µL) were added and incubated at 37 °C in 5% CO_2_ for 6 h. For the control, 10 µL of Dulbecco’s PBS (Thermo Fisher Scientific, Waltham, MA, USA) was used instead of MVs. Cell lysis and reverse transcription reactions were performed using the CellAmp^TM^ Direct TB Green RT-qPCR Kit (TaKaRa Bio Inc., Shiga, Japan). The real-time PCR reaction was performed using Thermal Cycler Dice^®^ Real-Time System II (TaKaRa Bio Inc., Shiga, Japan). The glyceraldehyde 3-phosphate dehydrogenase (GAPDH) gene was used as an internal standard to correct differences in the amount of mRNA between samples. The fold change value with a logarithm transformation [log 2 (2^–ΔΔCt^)] was calculated. The sequences of the primers are shown in Table 2.

### 2.11. Statistical Analysis

The gene expression results were expressed as the mean ± standard error (SE), and the other experimental results were expressed as the mean ± standard deviation (SD). 

The difference between groups was analyzed using one-way ANOVA with Dunnett’s multiple comparison post hoc tests and one-way ANOVA with Tukey’s post hoc test. The level of statistical significance was set at *p* < 0.05. Significant letters of the alphabet represent significant differences.

## 3. Results

### 3.1. Gene Expression of Virulence Factors of the SEA-Producing Strain

**Figure 1:**
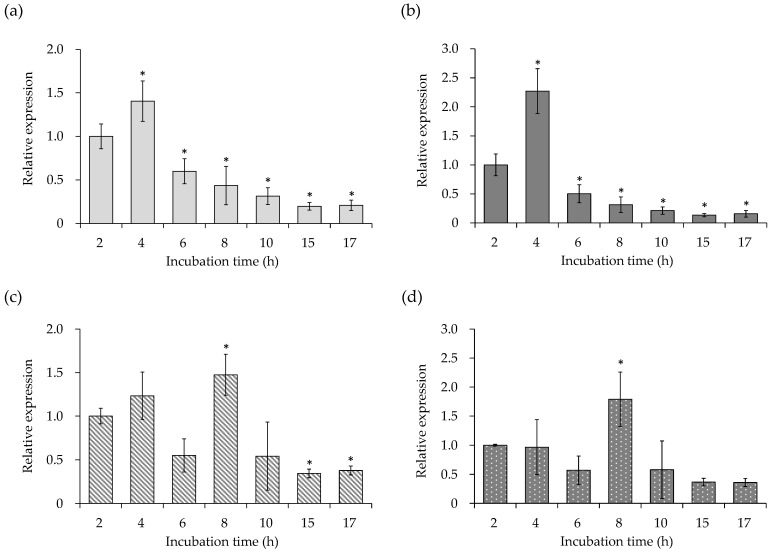
Time-dependent changes in the expression level of the virulence factor of *Staphylococcus aureus* C-29. Gene expression of (**a**) staphylococcal enterotoxin A (*sea*), (**b**) RNAIII, (**c**) phenol-soluble modulin α1 (*psm*α1), and (**d**) *psm*α3. *S. aureus* C-29 (10^8^ cells/mL) was inoculated into BHI and incubated at 37 °C with shaking. Bacterial cells were collected after 2, 4, 6, 8, 10, 15, and 17 h. Then, RNA extraction and real-time RT PCR were performed. The data are expressed as the mean ± SE of three or more independent experiments. * Represents *p* < 0.05 compared to 2 h.

The changes in the expression levels of virulence factors, such as SEA gene (*sea*), quorum sensing control gene (RNAIII), and PSM gene (*psmα*1 and 3) of *S. aureus* C-29, were examined. As shown in Figure 1, *sea* (Figure 1a) and RNAIII (Figure 1b) expression increased significantly during the exponential phase of 4 h. The expression of *psmα*1 (Figure 1c) and *psmα*3 (Figure 1d) decreased once over 6 h and increased significantly at 8 h.

### 3.2. Cell Membrane-Related Gene Expression of the SEA-Producing Strain

**Figure 2:**
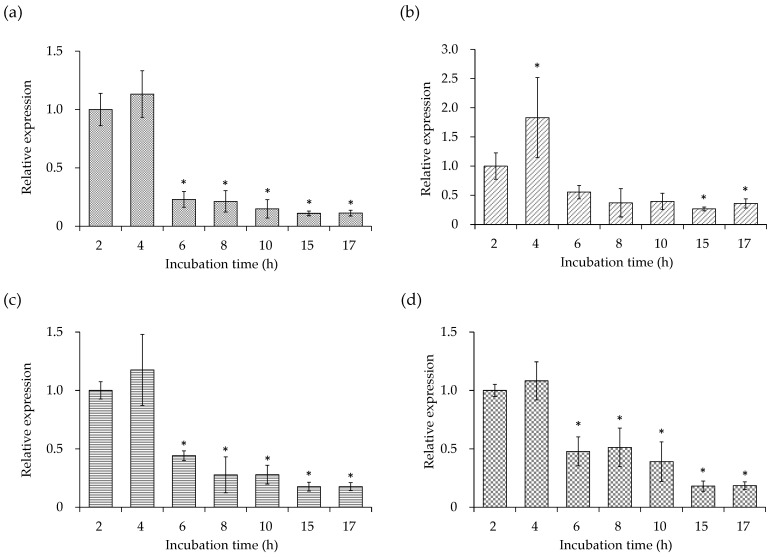
Time-dependent changes in the expression levels of cell membrane-related genes of *Staphylococcus aureus* C-29: (**a**) poly(glycerophosphate chain) *d*-alanine transfer protein (dltD) gene (*dlt*D), (**b**) multiple peptide resistance factor (mprF) gene (*mpr*F), (**c**) phosphatidylglycerol synthase (pgsA) gene (*pgs*A), and (**d**) cardiolipin synthase (cls2) gene (*cls*2). *S. aureus* C-29 (10^8^ cells/mL) were inoculated into BHI and incubated at 37 °C with shaking. Bacterial cells were collected after 2, 4, 6, 8, 10, 15, and 17 h. Then, RNA extraction and real-time RT PCR were performed. The data are expressed as the mean ± SE of three or more independent experiments. * Indicates *p* < 0.05 compared with 2 h incubation time.

The changes in the expression levels of cell membrane-related genes, such as *dlt*D, *mpr*F, pgsA gene (*pgs*A), and cls2 gene (*cls*2) of *S. aureus* C-29 were examined. As shown in Figure 2, the expression levels of all genes were decreased at 6 h compared to 2–4 h. Based on these gene expression patterns, MVs were prepared from the supernatant of the SEA-producing strain cultured at 37 °C for 6 (log phase), 17 (stationary phase), and 24 (phase of decline) h.

### 3.3. Particle Size Distribution of MVs

**Figure 3:**
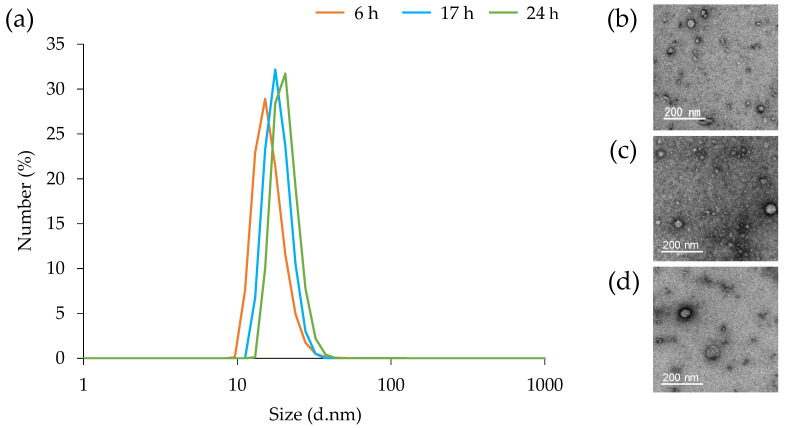
Particle size distribution of membrane vesicles (MVs) derived from *Staphylococcus aureus*. (**a**) The particle size distribution of MVs prepared from the culture supernatant after incubation for 6, 17, and 24 h. The average size distribution of the MVs was determined using dynamic light scattering. TEM images of MVs prepared from culture supernatants after incubation for 6 (**b**), 17 (**c**), and 24 h (**d**).

The particle size of MVs at each growth stage (6, 17, and 24 h) were measured using DLS and TEM. The particle diameter (Figure 3a) and morphology (Figure 3b–d) of MVs were essentially similar, regardless of incubation time. A size distribution range with a diameter of approximately 10–50 nm was found, with an average diameter of 18.2 nm. The mean protein concentrations were 12.89, 12.63, and 13.16 mg/mL after incubation for 6, 17, and 24 h, respectively. In summary, the incubation time did not affect the morphology, diameter and protein concentration of MVs.

### 3.4. Storage Stability of MVs

**Figure 4:**
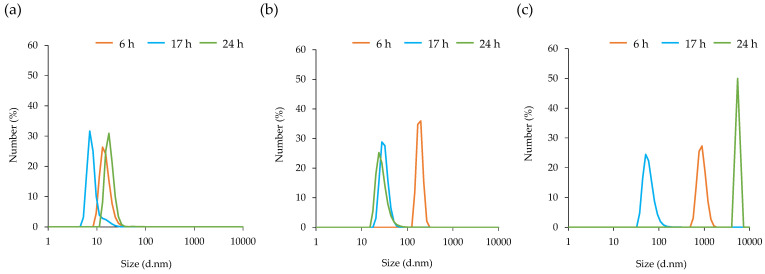
Stability of membrane vesicles (MVs) derived from *Staphylococcus aureus*. MVs prepared from the supernatant after culture for 6, 17, and 24 h were stored at 23 °C for (**a**) 2 days, (**b**) 5 days, and (**c**) 1 week. The average size distribution of the MVs was determined using dynamic light scattering.

The MVs prepared for each incubation time were stored at 23 °C, and the particle size was measured after 0, 2, and 5 days, and 1 week. TEM images of MVs after storage at 2, 5 days, and 1 week were shown in Supplementary Figure S1. From 0 days (Figure 3a) to 2 days (Figure 4a), there was no change in the diameter of the MVs depending on each growth stage. After 5 days of storage, the particle size of MVs prepared after incubation for 6 h was enlarged (Figure 4b). After 1 week of storage, the particle size of MVs was prepared by incubation for 6 and 24 h (Figure 4c). MVs prepared with incubation for 17 h had maintained particle size.

### 3.5. Amount of SEA in Culture Supernatant and MVs

**Figure 5:**
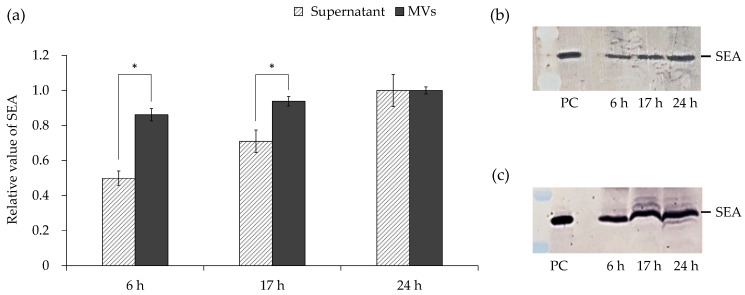
Comparison of the amount of SEA in the culture supernatant and membrane vesicles (MVs) derived from *Staphylococcus aureus*. (**a**) The relative value of SEA content in the culture supernatant and MVs. (**b**) The culture supernatant after different incubation periods (6, 17, and 24 h) and (**c**) corresponding MVs were analyzed by SDS-PAGE and visualized by Western blotting analysis. PC: positive control purified SEA. * Represents *p* < 0.05 compared to 24 h. Values represent the mean ± SD of three independent experiments.

The culture supernatants and inclusion proteins were compared in MVs at different growth stages. The amount of SEA in the supernatant obtained after incubation for 24 h was considered to be 100%, and the SEA amount was approximately 50% and 70% at 6 and 17 h, respectively (Figure 5a,b). In contrast, when the amount of SEA in MVs obtained after incubation for 24 h was 100%, the amount of SEA was approximately 86% and 94% at 6 and 17 h, respectively (Figure 5a,c). Hence, these results show that the amounts of SEA in the culture supernatant and MVs were not correlated.

### 3.6. Cargo Protein Composition in MVs

**Figure 6:**
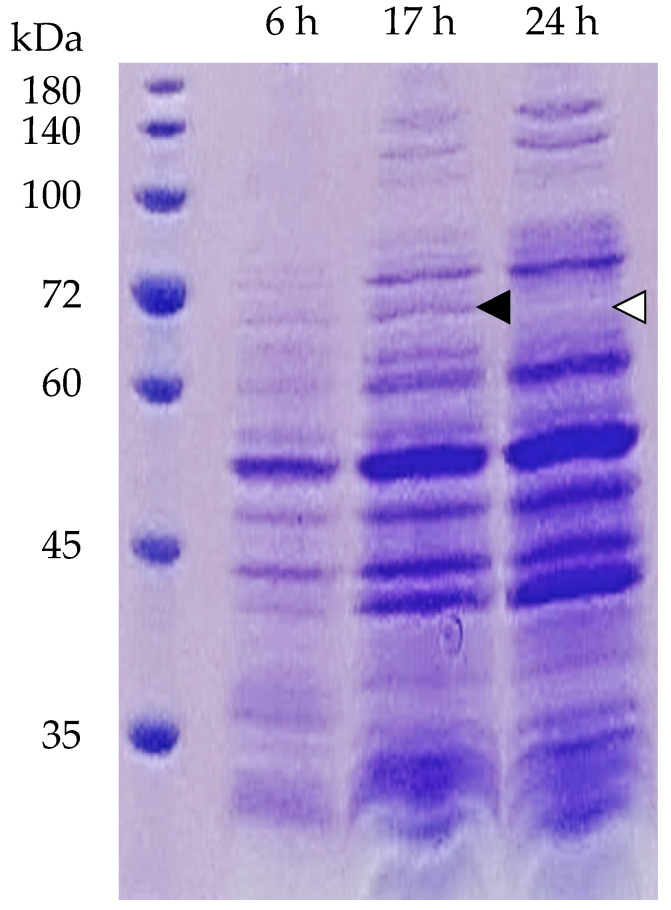
Cargo proteins in membrane vesicles (MVs) obtained from supernatants after different incubation times. MVs prepared from culture supernatants after incubation for 6, 17, and 24 h. Each cargo protein in the MVs was analyzed by SDS-PAGE. The arrows indicate the expression of protein bands that were upregulated (black) or downregulated (white).

A proteomic approach with SDS-PAGE and nano-LC–MS/MS analysis was used to reveal differences in the cargo protein content in MVs subjected to different incubation times. The arrows shown in Figure 6 indicate the expression of upregulated (black) or downregulated (white) protein bands. This one protein band was excised and identified using nano-LC–MS/MS analysis. Consequently, six proteins were identified by the MASCOT search engine (http://www.matrixscience.com, accessed on 12 January 2021). These proteins were extracted from the identified peptides with high reliability. As shown in Table 3, three proteins related to glycolysis, dihydrolipoyllysine-residue acetyltransferase-component of pyruvate dehydrogenase complex, enolase and dihydrolipoyl dehydrogenase, were estimated. In addition, formate acetyltransferase related to carbohydrate metabolism and CTP synthase related to pyrimidine and biosynthesis were estimated. As a protein involved in cell wall biosynthesis/degradation, bifunctional autolysin was estimated.

### 3.7. MV-Induced Inflammatory Mediator Release

**Figure 7:**
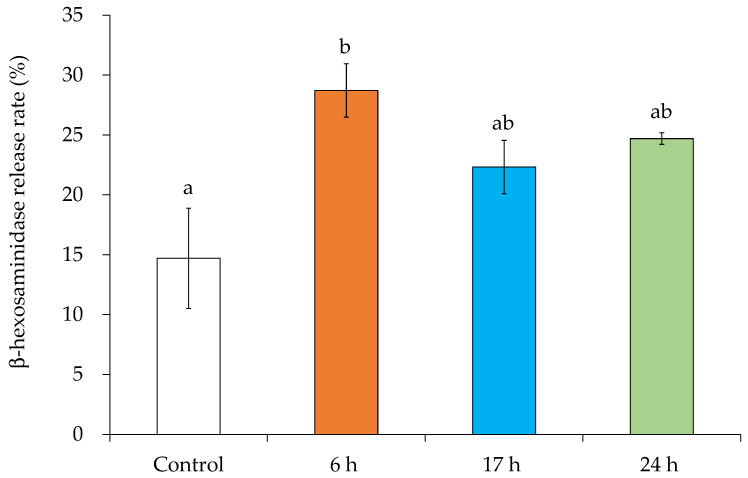
β-Hexosaminidase release induced by membrane vesicles (MVs) obtained from supernatants with different incubation times. MVs were exposed to IgE sensitized rat basophilic leukemia (RBL-2H3) cells for 1 h, followed by a β-hexosaminidase-release assay. Significant letters represent significant differences (Tukey–Kramer test, *p* < 0.05). Values represent the mean ± SD of three independent experiments.

The effect of MVs at different growth stages on inflammatory mediator release in RBL-2H3 cells was examined. In this study, the release of β-hexosaminidase, an inflammatory mediator, was measured. β-Hexosaminidase, similar to histamine, is used as an indicator of mast cell degranulation [31]. MVs increased the release of β-hexosaminidase from anti-DNP BSA-IgE sensitized RBL-2H3 cells. To compare to the DNP BSA-stimulated control group (14.7%), β-hexosaminidase release levels when treated with MVs obtained after incubation for 6, 17, and 24 h were 28.7%, 22.3%, and 24.7%, respectively (Figure 7). Different incubation times did not affect the release rate of β-hexosaminidase.

### 3.8. Expression Level of MV-Induced Inflammation-Related Genes

**Figure 8:**
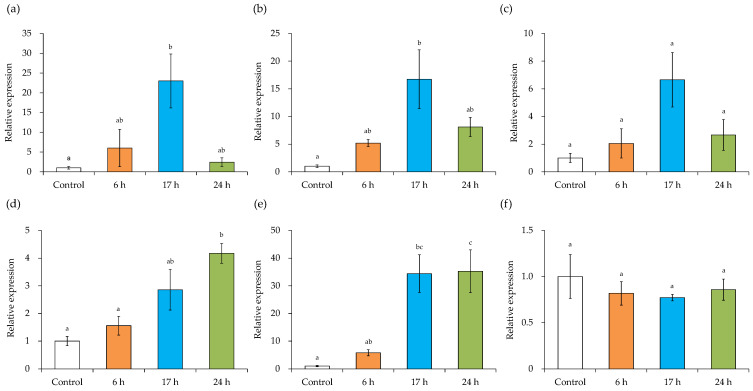
Expression levels of inflammation-related genes induced by membrane vesicles (MVs) obtained at different growth stages. Human adult low calcium high temperature (HaCaT) cells were incubated with MVs obtained at different growth stages (6, 17, and 24 h) derived from *S. aureus* for 6 h and the expression of inflammation-related genes was evaluated. (**a**) interleukin (IL)-1β, (**b**) IL-8, (**c**) tumor necrosis factor (TNF)-α, (**d**) IL-6, (**e**) monocyte chemoattractant protein-1 (MCP-1), and (**f**) phosphatase and tensin homolog deleted on chromosome 10 (PTEN). Different letters indicate significant differences (Tukey–Kramer test, *p* < 0.05). Values represent the mean ± SE of three independent experiments.

The effect of MVs at different growth stages on the expression level of inflammation-related genes in HaCaT cells was examined. The mRNA expression of IL-1β (Figure 8a) and IL-8 (Figure 8b) was significantly increased in MVs obtained after incubation for 17 h, as compared with the control without MVs. The mRNA expression of TNF-α (Figure 8c) also tended to increase in MVs obtained after incubation for 17 h. The mRNA expression of IL-6 (Figure 8d) and MCP-1 (Figure 8e) was significantly increased in MVs obtained after incubation for 17 h and 24 h, as compared with the control. The mRNA expression of PTEN (Figure 8f) followed a decreasing tendency in MVs from all growth stages as compared with control.

## 4. Discussion

When the changes in the gene expression of *S. aureus* C-29 at different growth stages were examined, it was found that the mRNA expression was maximized at 4 h for *sea* (Figure 1a) and RNA III (Figure 1b), and at 8 h for psm*α*1 and 3 (Figure 1c,d). PSM causes the leakage of *S. aureus* membranes and can induce the release of cytoplasmic proteins [32]. Furthermore, in recent years, PSM was reported to be involved in the release and destruction of MVs [13]. The PSM export system Pmt exists because PSM accumulates in the bacterial cytosol and causes substantial inhibition of growth [33]. It was suggested that the Pmt ABC transporter removes the PSM from the membrane and excretes it as a free molecule, releasing the MV containing the PSM. PSM and most SEs are controlled by the *agr* system, and they are synergistically co-generated in the late exponential phase [34,35]. As SEA is not controlled by the *agr* system [5,6,7], it was suggested that the time-course of the expression patterns of PSM and SEA was different.

Next, the changes in the expression levels of the four cell membrane-related genes (*dlt*D, *mpr*F, *pgs*A, and *cls*2) of *S. aureus* C-29 at different growth stages were examined. As a result, the expression levels of all cell membrane-related genes were decreased at 6 h compared to 2–4 h (Figure 2). In Gram-positive bacteria, the cell envelope is composed of the cell membrane and the cell wall peptidoglycan layer, and the protein and teichoic acid (TA) are covalently bound. The d-alanylation of TA requires an operon containing *dlt*ABCD [36]. The esterification of d-alanine to TA regulates the activity of the self-degrading enzymes [37,38], and binds cations to the cell envelope [39,40,41], resulting in resistance to many antibacterial cationic peptides/proteins [42,43,44]. Lysylphosphatidylglycerol (LPG), a major component of the cell wall of *S. aureus* [45], is a basic lipid in which the lysyl group of lysyl tRNA is transferred to PG [46,47,48]. The MprF protein functions as an LPG synthetase and reduces the negative charge on cell membranes [49]. Further, pgsA and cls2 are enzymes involved in the synthesis of the anionic membrane lipids, PG and CL [23,50,51]. PG is processed to L-PG by mprF, and CL by Cls1/Cls2 [52]. An increased negative charge on the cell membranes accelerates autolytic activity. In this study, the genes required for *S. aureus* to synthesize the cell membrane were highly expressed during the growth phase of culture (6 h), which may affect the membrane state of MVs. It was suggested that the activity of the autolytic enzymes (autolysins) increased after 6 h when the expression level of *dlt*D, *mpr*F, *pgs*A, and *cls*2 decreased significantly. Changes in the expression levels of these genes may contribute to the stability of MVs prepared at each growth stage. Since the expression levels of *psm* showed a maximum after 8 h (Figure 1c,d), and the four cell membrane-related genes (*dlt*D, *mpr*F, *pgs*A and *cls*2) decreased in 6 h (Figure 2), MVs were prepared at 6 (log phase), 17 (stationary phase), and 24 (phase of decline) h of incubation.

Factors influencing peptidoglycan cross-linking, such as autolysine, affect the size of MVs because they control the ability of MVs to penetrate the cell wall [53]. The particle size distribution of MVs derived from *S. aureus* C-29 did not change with the incubation time (Figure 3). It was suggested that PSMα3 promotes the release of MVs from the cytoplasmic membrane of *S. aureus*, but does not affect their size. MVs at each incubation time were stored at 23 °C for 0, 2, and 5 days, and 1 week, and their particle size was measured. The particle size was maintained for 2 days in all MVs (Figure 4a). After 5 days, the particle size of only MVs prepared after 6 h of incubation was enlarged (Figure 4b). After 1 week, MVs prepared after 6 and 24 h of culture tended to shrink or expand (aggregate), and MVs prepared after 17 h of culture maintained particle size (Figure 4c). PSMα3 itself is contained in MVs; at high concentrations, it induces MVs destruction [13]. A large amount of PSM was present in the MVs after being prepared for 17 h, which may be involved in the destruction of the MVs. Differences in cell membrane composition may be involved in the differences in MV stability. The bacteria that contain mycolic acids were reported to release MVs when the concentration of biotin, which is essential for cell membrane synthesis, is low [54]. After 6 h of culturing, it is considered that the medium contains nutrients that are essential for the synthesis of cell membranes. After incubation for 6 h, it was considered that the medium contains nutrients that are essential for the synthesis of cell membranes. There were differences in cell membrane components after 6 and 17 h of incubation; hence, it was possible that MVs are released by different routes.

Cargo proteins in MVs at different growth stages were compared. After 6 h of culture, the amount of SEA in the culture supernatant was smaller than that in 24 h (Figure 5a,b); in contrast, for MVs, there was no significant difference in the amount of SEA between 6 h and 24 h (Figure 5a,c). These results indicate that SEA may be selectively included in MVs in the bacteria incubated for 6 h. It was reported that the major virulence factors of *Porphyromonas gingivalis* (mainly gingipain) are preferentially packaged in MVs [55]. Gram-negative bacteria utilize lipopolysaccharides, a component of the outer cell wall membrane, to cause preferential packing of selected proteins into MVs. However, little is known about the biosynthesis and composition of MVs in Gram-positive bacteria compared with Gram-negative bacteria [10,56]. SEA, along with other SEs, is mostly attributed to food poisoning, which is believed to be due to its high resistance to proteolytic enzymes [57]. In this study, it was clarified that SEA was contained at a high concentration in MVs after incubation for 6 h. If MVs contain high concentrations of SEA, the ingestion of small amounts of food can cause food poisoning.

Differences in the proteins included in MVs obtained at each growth stage were examined. Increases in some proteins were detected that increased in MVs obtained at 17 h incubation and decreased in MVs obtained at 24 h incubation (Figure 6). Bifunctional autolysin was included in these proteins (Table 3). Bifunctional autolysin has peptidoglycan hydrolyzing activity and is involved in cell wall turnover, cell separation, cell division, and agonist-induced autolysis [58]. The modification of the cell wall is believed to be an important process in the formation of MVs in Gram-positive bacteria [59]. Endoricin [53], a peptidoglycan hydrolase, autolysin was suggested to induce MVs formation in *S. aureus* [60]. This study showed that bifunctional autolysin is present in *S. aureus*-derived MVs. The increase in bifunctional autolysin increased cell wall autolysins, suggesting that MV release was promoted after incubation for 17 h.

In allergic reactions, antigen binding to the high-affinity IgE receptor (FcεRI) on the surface of mast cells and basophils induces the release of intragranular mediators, such as histamine, serotonin, and β-hexosaminidase [61,62]. In rat basophilic leukemia RBL-2H3 cells, IgE bound to the cell surface is cross-linked by an antigen, and granulocytes, such as histamine, are released extracellularly. The β-hexosaminidase assay has been widely used to monitor the degranulation of RBL-2H3 cells [31,63]. The exposure of MVs to RBL-2H3 cells significantly increased the release rate of β-hexosaminidase, regardless of incubation time (Figure 7). The MVs used in this study did not induce cell proliferation suppression or cytotoxicity (data not shown). Staphylococcal enterotoxin B (SEB) can directly cause degranulation in RBL-2H3 cells [64]. Little is known about the direct effects of SEA on basophils. In this study, the stimulation of RBL-2H3 cells with SEA, similar to SEB, did not induce the release of β-hexosaminidase, an indicator of degranulation (data not shown). Basophils in patients with atopic dermatitis, who have a high carrier rate of *S. aureus*, release histamine via the FcεRI receptor [65]. Therefore, a molecule other than SEA may induce inflammation of mast cells and basophils. *S. aureus*-derived MVs can directly cause mast cell degranulation and exacerbate allergy symptoms. The induction of allergic inflammation favors *S. aureus*. Immune regulation by *S. aureus* may increase the risk and severity of bacterial infection.

The effects of MVs at different growth stages on the expression level of inflammation-related genes of HaCaT cells were examined. The mRNA expression of IL-1β (Figure 8a), IL-8 (Figure 8b), and TNF-α (Figure 8c) was most increased in MVs obtained by incubating for 17 h. *S. aureus* MVs containing staphylococcal protein A (SPA) stimulated the expression of keratinocyte pro-inflammatory cytokine genes, including IL-1β and IL-8 in vitro [25]. Therefore, SPA in MVs may affect the induction of inflammation in keratinocytes. The amount of SPA in the culture supernatant was expected to be higher after 24 h, but MVs obtained by incubation for 17 h increased the mRNA expression of IL-1β (Figure 8a), IL-8 (Figure 8b), and TNF-α (Figure 8c) after more than 24 h. In contrast, the mRNA expression of IL-6 (Figure 8d) and MCP-1 (Figure 8e) was most increased in MVs obtained after incubation for 24 h. It has been reported that the mRNA expression of IL-8, IL-6, and MCP-1 was significantly upregulated in HaCaT cells after treatment with SEA [66]. An increase in the mRNA expression of IL-8, IL-6, and MCP-1 may have been induced by SEA in MVs. It has been reported that the expression of the IL-6 gene was significantly increased with intact MVs than the disrupted MVs [25]. Furthermore, thymol suppresses MV-induced inflammation of host cells by disrupting *S. aureus*-derived MVs [26]. MVs interact with the membrane microdomain in the cytoplasmic membrane of host cells to deliver MV components [67]. The stability of MVs may be involved in the difference in the ability to induce inflammation-related genes.

The mRNA expression of PTEN (Figure 8f) followed a decreasing trend in MVs at all stages of growth compared with the control. It has been reported that PTEN was downregulated in IL-22-stimulated HaCaT cells [68]. The interaction of IL-22 with its receptors IL-22R/IL-10R2 induced skin inflammation by regulating the expression of numerous cytokines (e.g., IL-1β, TNF-α, IL-6, etc.) and chemokines [69,70]. It has also been reported that IL-22 may directly activate phospholipase C epsilon (PLCε), which is essential for the regulation of cytokine production in various types of skin inflammation in keratinocytes [71]. In this study, MVs tended to reduce PTEN expression, so the involvement of IL-22 and the subsequent signaling pathways should be examined.

There are still some limitations to this study. MVs samples from three different growth stages were normalized by the amount of culture supernatant, but not by the number of MVs. The difference in the inflammation-inducing ability of MVs in the three different growth stages may be due to the different number of MVs. In addition, MVs are not purified. It has been reported that the MVs-precipitation reagent (ExoQuick-TC; System Biosciences, Palo Alto, CA, USA) can isolate and purify MVs as well as density gradient ultracentrifugation (OptiPrep) [13]. However, in the MVs prepared by the ExoQuick-TC kit, not only MVs but also other aggregates were confirmed on the TEM image, and they were not electrophoresed well by SDS-PAGE (Supplementary Figure S2a,b). Therefore, MVs were prepared by the ultrafiltered and ultracentrifugal methods used. The culture supernatant was ultrafiltered, and most of the proteins in *S. aureus* culture filtrates were found in the flow through (<100 kDa) fraction. In addition, regarding the MVs-induced degranulation reaction, similar results were obtained between the MVs prepared by the kit and the MVs prepared by the ultracentrifugation method (Supplementary Figure S2c). Therefore, it is considered that there are few proteins that affect the data in MVs preparations. Moreover, further research is needed to confirm the molecules involved in inflammation induction in MVs. Understanding the role of MVs may lead to the development of new preventive and therapeutic tools for food poisoning and disease caused by *S. aureus* and SEs.

## 5. Conclusions

In this study, to demonstrate that the properties of MVs may differ at each growth stage, MVs were prepared at 6, 17, and 24 h. In summary, our study revealed that the growth stage affects the inclusion components of MVs from *S. aureus*. The SEA content of MVs remained almost unchanged, whereas the SEA was lower in the culture supernatant collected during the logarithmic growth phase (6 h) compared with the stationary phase (17–24 h). *S. aureus*-derived MVs from different growth stages had a similar inflammation-inducing ability to select the host cell responses, despite the difference in inclusion components. Owing to the high concentration of SEA contained in the MVs released during the logarithmic growth phase, there may be a large amount of staphylococcal food poisoning by induced SEA. MVs at different growth stages showed different inflammation-inducing patterns in HaCaT cells. Further research is expected to elucidate various unknown factors that influence the selective uptake of virulence factors in MVs.

## Figures and Tables

**Table 1:** The sequences of the primers used for the gene expression of *Staphylococcus aureus* C-29.

Gene	Forward Primer (5′ to 3′)	Reverse Primer (5′ to 3′)
16S rRNA	CGTGCTACAATGGACAATACAAA	ATCTACGATTACTAGCGATTCCA
*sea*	GATCAATTTATGGCTAGACG	CGAAGGTTCTGTAGAAGTATGA
RNAIII	CGATGTTGTTTACGATAGCTT	CCATCCCAACTTAATAACCA
*psm*α1	TATCAAAAGCTTAATCGAACAATTC	CCCCTTCAAATAAGATGTTCATATC
*psm*α3	CATTCACATGGAATTCGTAGCA	TCGTTTTGTCCTCCTGTATGTTG
*dlt*D	TGACCCATTTAATCCTGCAATTG	TCTGTAGAACCACCAGCACCTAATAA
*mpr*F	TTGTAGGTTTCGGTGGCTTT	GATGCATCGAAAACATGGAA
*pgs*A	TGGCTTCCCTTAGCGATTTTGT	CAGTTACGGCAAATTCTCTGGC
*cls*2	ATTAGAGTTAATCGTTGATGAGCAAT	TTACGGATGTCTTGTATTAGGTCAT

**Table 2:** The sequences of the primers used for the gene expression of membrane vesicle (MV)-induced genes in HaCaT cells.

Gene	Forward Primer (5′ to 3′)	Reverse Primer (5′ to 3′)
GAPDH	GGACCTGACCTGCCGTCTAG	GAGGAGTGGGTGTCGCTGTT
IL-1β	CCTGTCCTGCGTGTTGAAAGA	GGGAACTGGGCAGACTCAAA
IL-8	TTGGCAGCCTTCCTGATTTC	TGGTCCACTCTCAATCACTCTCA
TNF-α	CCCAGGGACCTCTCTCTAATC	ATGGGCTACAGGCTTGTCACT
IL-6	TGGCTGAAAAAGATGGATGCT	TCTGCACAGCTCTGGCTTGT
MCP-1	TCGCTCAGCCAGATGCAAT	TGGCCACAATGGTCTTGAAG
PTEN	GTTTACCGGCAGCATCAAAT	CCCCCACTTTAGTGCACAGT

**Table 3:** Cargo proteins in MVs with changed expression level after different incubation times.

Biological Process	Protein	MW	Score	Coverage	Accession
Glycolysis	Dihydrolipoyllysine-residue acetyltransferase-component of pyruvate dehydrogenase complex	46,395	598	36	Q6GHZ0
Carbohydrate metabolism	Formate acetyltransferase	84,862	500	21	Q2G1D8
Glycolysis and virulence	Enolase	47,117	427	21	O69174
Pyrimidine biosynthesis	CTP synthase	59,992	164	7	P65924
Glycolysis	Dihydrolipoyl dehydrogenase	49,451	148	11	Q6GAB8
Cell wall biogenesis/ degradation	Bifunctional autolysin	137,528	134	3	Q6GI31

## Data Availability

The data presented in this work are available in insert article.

## Supplementary Materials

The following supporting information can be downloaded at: https://www.mdpi.com/article/10.3390/microorganisms10030574/s1, Figure S1: TEM images of MVs stored at 23 °C for 2, 5 days, and 1 week, Figure S2: Cargo proteins in MV prepared using MVs-precipitation reagent (ExoQuick-TC).
