# Supplementary material for: Effects of Growth Stage on the Characterization of Enterotoxin A-Producing Staphylococcus aureus-Derived Membrane Vesicles
Source: Microorganisms. 2022 Mar 6;10(3):574. doi: 10.3390/microorganisms10030574 (PMC8948643; doi:10.3390/microorganisms10030574)
Supplement: Supplementary file 1 [file microorganisms-10-00574-s001.zip › microorganisms-1584488-supplementary.pdf]

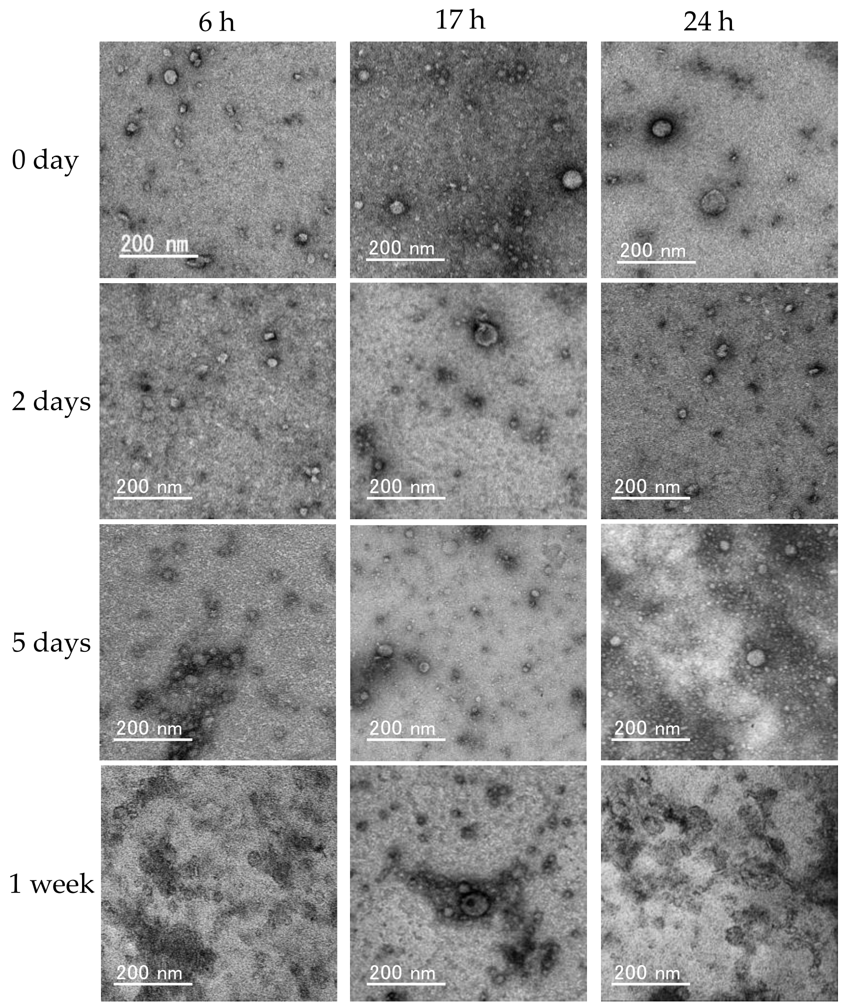

Figure S1. TEM images of MVs stored at 23°C for 2, 5 days, and 1 week. MVs prepared from culture supernatants after incubation for 6, 17, and 24 h.

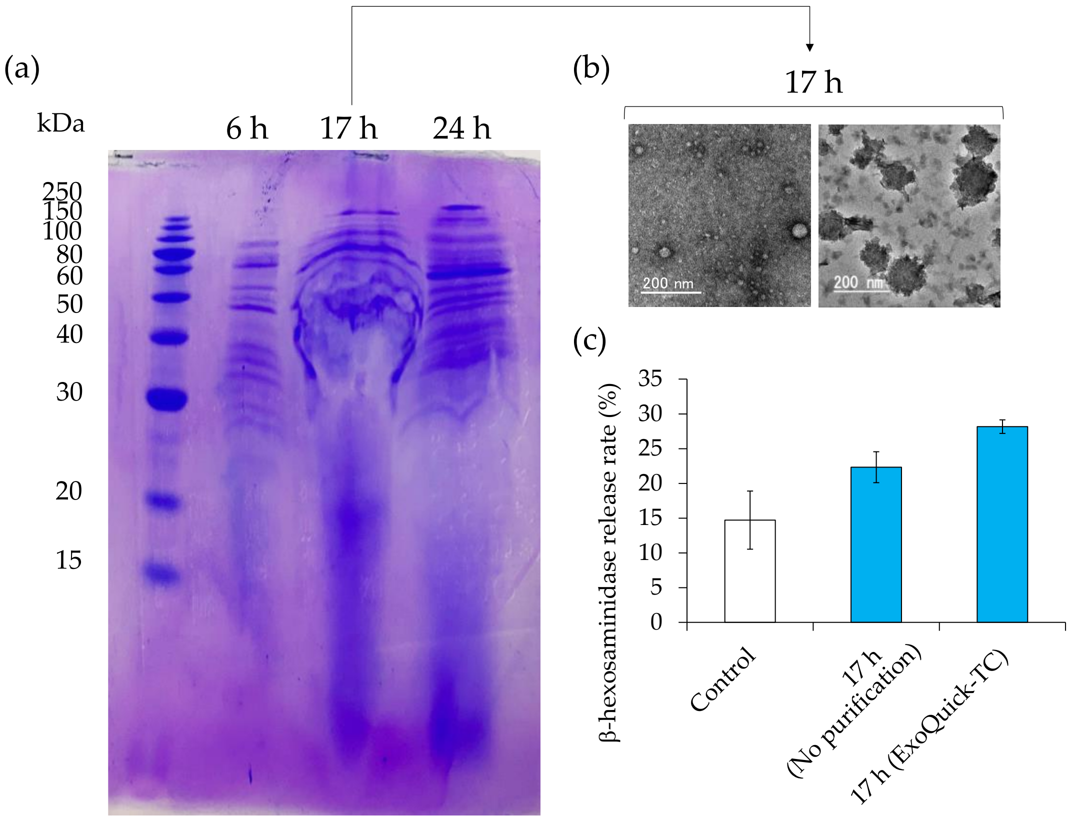

Figure S2. Cargo proteins in MV prepared using MVs-precipitation reagent (ExoQuick-TC). (a) MVs prepared from culture supernatants after incubation for 6, 17, and 24 h was analyzed by SDS-PAGE. (b) TEM images of MVs prepared using ExoQuick-TC from culture supernatants after incubation for 17 h. (c) β-Hexosaminidase release induced by MVs prepared using ExoQuick-TC.
